# Patch Antenna Design and Experimental Validation for Biomedical IoT Communication in 2.4 GHz ESP32-Based Health Monitoring Systems

**DOI:** 10.3390/s26123841

**Published:** 2026-06-17

**Authors:** Younes Siraj, Youssef Khardioui, Youssef Mejdoub, Hela Elmannai, Jaouad Foshi, Mohammed El Ghzaoui

**Affiliations:** 1 ISMSE Laboratory, Faculty of Sciences and Technology, Errachidia, Moulay Ismail University of Meknes, Meknes 50050, Morocco; 2 Laboratory of Networks, Computer Science, Telecommunication & Multimedia (RITM), Higher School of Technology, Hassan II University, Casablanca 20360, Morocco; 3 Department of Information Technology, College of Computer and Information Sciences, Princess Nourah bint Abdulrahman University, P.O. Box 84428, Riyadh 11671, Saudi Arabia; 4 Faculty of Sciences, Sidi Mohamed Ben Abdellah University, Fez 30000, Morocco

**Keywords:** patch antenna, biomedical, ESP32, Blynk, DGS, IOT, health monitoring

## Abstract

This paper presents a compact wearable patch antenna operating in the 2.4 GHz ISM band for biomedical Internet of Things (IoT)-based healthcare monitoring applications. The proposed antenna is intended for integration with wearable biomedical sensors in order to support real-time physiological data transmission in remote patient monitoring systems. The antenna was designed on an FR4 substrate to achieve good impedance matching and stable radiation performance. The antenna showed good performance, with a reflection coefficient of −39.56 dB and a gain of 3.01 dB. SAR analysis confirmed compliance with IEEE and ICNIRP safety standards for wearable applications. In addition, the antenna prototype was fabricated and experimentally validated using a vector network analyzer (VNA), showing good agreement between simulated and measured results. Furthermore, the proposed system was implemented by integrating an ESP32 microcontroller with a MAX30100 physiological sensor, where the sensor is responsible for acquiring real-time biomedical data, including heart rate and blood oxygen saturation (SpO_2_). The ESP32 processes the acquired data and enables wireless transmission through the proposed antenna to a smartphone and laptop using the Blynk IoT platform, which allows real-time remote monitoring and visualization of physiological parameters. The obtained results confirm the suitability of the proposed antenna for wearable biomedical devices, remote healthcare monitoring, and IoT-enabled healthcare applications.

## 1. Introduction

Wireless communications have witnessed many recent developments that have had a significant impact on the development of smart healthcare monitoring systems. Specifically, there is a growing demand for remote monitoring of patients, as well as for wearable medical devices and transmission of physiological data in real time [1,2]. Furthermore, compact, efficient, and reliable antennas that can operate in the industrial, scientific, and medical (ISM) frequency ranges are becoming increasingly necessary to support the creation of these systems [3,4]. In this context, patch antennas have received considerable attention because of being low profile, lightweight, easy to manufacture, and compatible with many modern wireless systems, including biomedical applications [5,6,7], sensing [8,9], imaging [10,11,12], and satellites [13,14].

The continued success of biomedical Internet of Things (IoT) systems is highly dependent on the ability to maintain a stable wireless connection at all times to allow for ongoing communication among the sensor, processing unit and monitoring platform. Many different wireless communication technologies are currently available, but Wi-Fi communication operating around 2.4 GHz remains the most widely adopted solution because of its accessibility, low implementation cost, and compatibility with embedded systems such as Espressif Systems ESP32 modules [15,16]. Consequently, the antennas designed for this type of application must have desirable characteristics, including a wide bandwidth and good radiation characteristics to support uninterrupted data transmission in practical environments.

Many recent works have investigated patch antennas for biomedical applications and wireless healthcare uses. However, most of the reported studies focused only on simulation analysis, without any validation of the antenna performance in real operating scenarios. In practical biomedical cases, the antenna performance is influenced by many factors, including fabrication tolerances, wireless communication stability, and integration with other sensing devices. Therefore, the experimental validation of fabricated antennas using actual biomedical monitoring systems has become very important to demonstrate the feasibility of the proposed antenna structures.

Many studies have reported the integration of compact antennas with biomedical IoT and wireless healthcare monitoring systems. In [17], the authors presented a battery-free, wireless, and skin-mountable multisensory patch for biosignal monitoring. The proposed system integrated ECG, SpO_2_, and temperature sensing with NFC energy harvesting and Bluetooth communication. However, the study mainly focused on short-range wearable monitoring and did not investigate the long-range communication performance. Similarly, ref. [18] proposed a wearable microwave-based patch antenna sensor for tumor detection by analyzing variations in the reflection coefficient (S_11_) caused by changes in tissue dielectric properties. The study demonstrated the potential of non-invasive microwave sensing for detecting tumors in different body parts using wearable antenna configurations. Nevertheless, the study mainly focused on sensing performance and did not investigate the integration of the antenna within a complete IoT healthcare platform. The authors of [19] presented a single-port substrate-integrated waveguide (SIW) resonator sensor designed for non-invasive blood glucose monitoring based on dual-parameter electromagnetic sensitivity. The system exploits variations in resonant characteristics to detect changes in the dielectric properties of biological tissues linked to glucose concentration. The work demonstrated high sensing potential for biomedical applications using compact SIW-based microwave structures, but full integration into a wearable IoMT system and SAR safety evaluation remain limited. Moreover, ref. [20] reported a compact, unified hexagonal dual-band wearable patch antenna designed for healthcare and body-centric communication systems. The antenna achieved satisfactory impedance matching and radiation characteristics in the targeted operating bands. Despite these advantages, the study mainly focused on antenna performance and did not demonstrate integration with a complete biomedical monitoring platform. Recently, ref. [21] investigated a printed slot antenna for non-invasive glucose sensing using an FR4 substrate, highlighting the growing interest in microwave antenna technologies for biomedical diagnostics. The proposed sensor exhibited promising sensitivity and demonstrated the feasibility of antenna-based glucose monitoring. However, the work primarily concentrated on sensing performance and did not include the implementation of a real-time wireless healthcare monitoring system. Motivated by these limitations, the present work proposes a compact circular slotted patch antenna with a defected ground structure operating at 2.45 GHz. Unlike previous studies, the proposed antenna combines compact size, excellent impedance matching, satisfactory radiation performance, SAR evaluation for wearable safety, fabrication and measurement validation, and integration with an ESP32-based biomedical IoT platform for real-time physiological data transmission, thereby providing a more comprehensive and practically deployable solution for wearable healthcare monitoring applications.

Unlike other reported studies that mainly focused on antenna simulation or partial system-level demonstrations, this work presents a compact circular slotted patch antenna with a defected ground structure (DGS) operating at 2.4 GHz for biomedical IoT applications. The proposed antenna was designed and optimized to achieve high impedance matching, a low reflection coefficient, and good radiation characteristics suitable for wearable and on-body communication environments. The proposed design was fabricated and experimentally validated, with the measured results showing good agreement with simulations, which confirmed the reliability of the antenna. The antenna performance was further evaluated in terms of the specific absorption rate (SAR) in order to ensure safe operation in close proximity to the human body. In addition, the proposed antenna was integrated into a complete real-time biomedical IoT system using an ESP32 microcontroller (Espressif Systems, Shanghai, China) and a MAX30100 physiological sensor (Maxim Integrated Products Inc., San Jose, CA, USA), which enable wireless transmission of heart rate and SpO_2_ data via Wi-Fi to the Blynk platform for remote monitoring through smartphone and computer interfaces, thereby demonstrating a fully validated end-to-end healthcare monitoring solution.

## 2. Patch Antennas in Biomedical Applications

**Figure 1:**
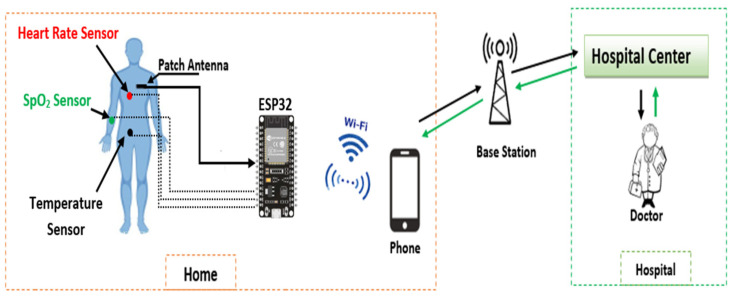
Application of patch antennas in biomedical monitoring.

Patch antennas are the most common antenna type used in biomedical applications because of their compact size, low profile, and ease of integration with wearable and wireless healthcare devices. In such applications, the antenna is generally positioned on or near the human body to maintain wireless communication between sensors and external monitoring systems, as presented in Figure 1. All physiological data collected from different sensors, like heart rate, oxygen saturation, or body temperature, are processed using embedded modules like ESP32 and transmitted through the patch antenna toward nearby devices, including smartphones or personal computers, using wireless communication technologies operating in the ISM band. Then, the data can be communicated via networks and base stations to the hospital center to allow doctors and staff members to monitor the current status of patients and provide rapid medical intervention in instances of abnormality or emergency.

## 3. Structure of the Proposed Antenna

**Figure 2:**
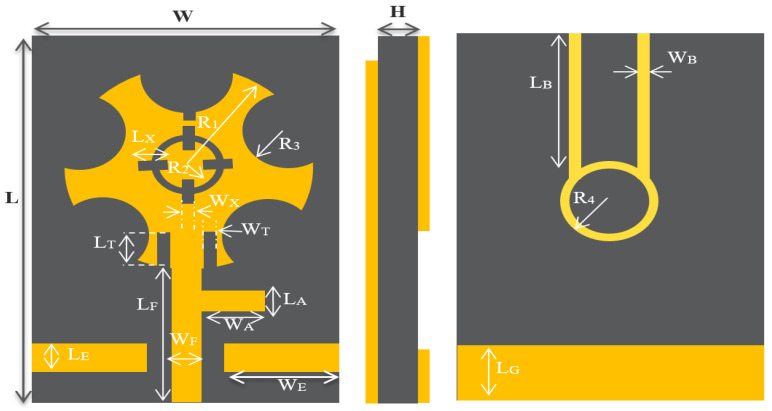
Structure of the proposed antenna.

The proposed antenna is a compact circular patch implemented on a substrate of FR4 characterized by a dielectric constant of 4.4 and a loss tangent of 0.02, with copper layers on the top and bottom sides, as presented in Figure 2. The radiating element, located on the front side, is excited using a microstrip feed line of 50 Ω. In order to improve the antenna performance in terms of impedance matching and bandwidth, many modifications are introduced into the radiating structure. The final design is a symmetrical flower-shaped geometry formed by five circular cuts with radius R_3_, while a circular ring of radius R_2_ is incorporated at the center of the main radiating element to improve the current distribution and electromagnetic coupling.

In addition, a rectangular stub with dimensions W_A_ and L_A_ is inserted and attached to the feed line in order to enhance the antenna impedance matching. Furthermore, two narrow slots are inserted between the feed line and the main patch structure to enhance the matching and minimize the reflected energy.

On the bottom side of the antenna, a partial ground plane with length L_G_ is used instead of a full ground plane. The defected ground structure (DGS) consists of two narrow rectangular strips with dimensions L_B_ and W_B,_ terminated by a circular ring with radius R_4_. This modified ground configuration contributes to bandwidth enhancement, impedance matching improvement, and stable radiation performance. The overall antenna dimensions are listed in Table 1.

**Figure 3:**
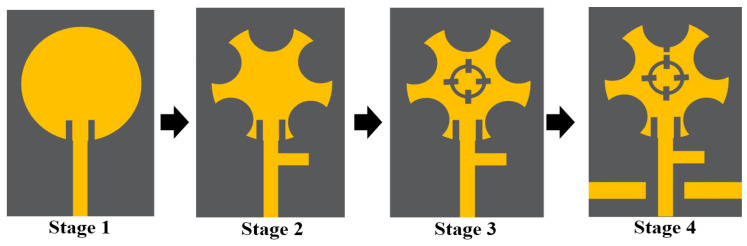
Antenna design process.

**Figure 4:**
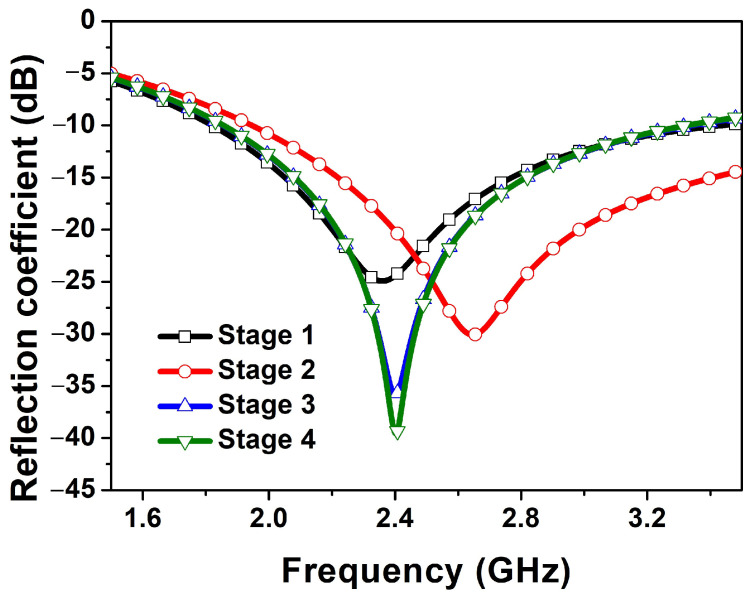
Reflection coefficient for antenna stages.

Figure 3 illustrates the design process of the proposed antenna, which consisted of four stages, while the corresponding S_11_ results are presented in Figure 4. Initially, Stage 1 was a conventional circular patch antenna, for which the first dimensions were calculated using (1) and (2). The antenna resonated near the desired frequency with poor impedance matching performance. In Stage 2, the radiating element was modified, which resulted in improved matching characteristics and a resonant frequency shift. Further enhancement was achieved in Stage 3 by the integration of additional slots at the center of the patch, which led to a reduction in the reflection coefficient near 2.4 GHz. Finally, Stage 4 included ground plane modifications and two parasitic elements to optimize the current distribution and impedance matching, which led to the best antenna performance, with a reflection coefficient close to −40 dB in the targeted 2.4 GHz ISM band.

(1) $$a=\frac{F}{\sqrt{1+\frac{2h}{F\pi \varepsilon_{r}}(ln\frac{\pi F}{2h}+1.7726)}}$$

(2) $$F=\frac{8.791\times 10^{9}}{f_{r}\sqrt{\varepsilon_{r}}}$$

**Figure 5:**
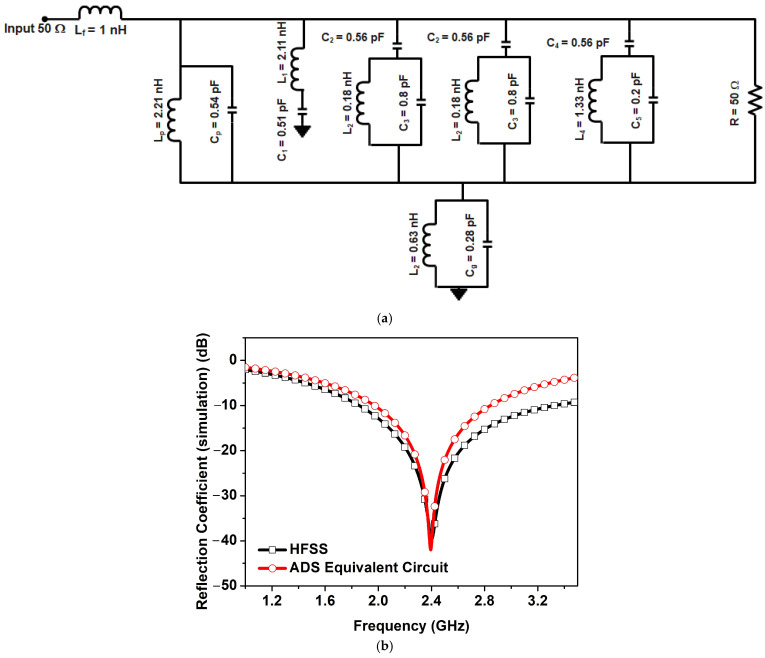
(**a**) Equivalent circuit of the proposed antenna; (**b**) S_11_ results obtained from HFSS and ADS.

The equivalent circuit of the proposed antenna is presented in Figure 5a. The model is a combination of lumped inductive and capacitive elements that represent the electromagnetic behavior of the antenna structure, including the patch, feed line, and defected ground plane. The values of the circuit components were optimized to match the resonant characteristics observed in the HFSS simulation. Figure 5b presents a comparison of the reflection coefficient obtained from HFSS and the equivalent circuit model simulated using Advanced Design System (ADS). A good agreement can be observed between the two results, particularly around the resonant frequency of 2.4 GHz, where both models show a deep resonance. This agreement between the results confirms the validity of the proposed equivalent circuit and demonstrates its effectiveness in representing the antenna’s impedance behavior while providing a simplified approach for antenna analysis and optimization.

## 4. Parametric Study

### 4.1. Feed Stub Length L_A_

**Figure 6:**
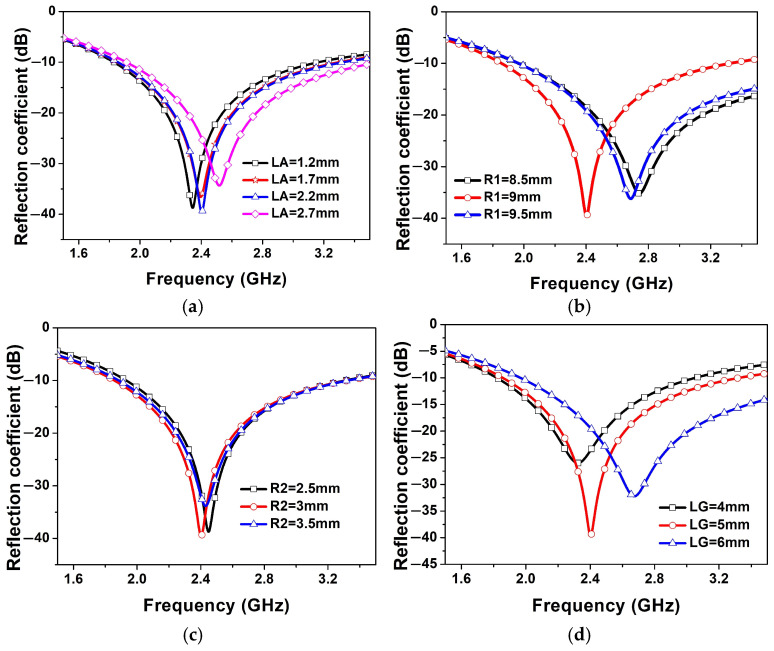
Reflection coefficient with different (**a**) L_A_ values, (**b**) R_1_ values, (**c**) R_2_ values, and (**d**) L_G_ values.

The influence of the parameter L_A_ on the antenna performance was investigated through a parametric analysis. Figure 6a presents the impact of the parameter on the reflection coefficient of the proposed antenna. It can be observed from the curve that the value of L_A_ affected both the resonant frequency and the impedance matching performance. The parameter was varied from 1.2 to 2.7 mm, with 0.5 increments. Among all tested values, L_A_ = 2.2 mm provided the best impedance matching with the lowest S_11_ of −39.56 dB at 2.4 GHz.

### 4.2. Radius R_1_ of Central Circular Ring

Another parametric study was performed by varying the radius of the central ring R_1_. Figure 6b presents the influence of this parameter on the antenna performance. It is evident that the variation in R_1_ strongly affected the center frequency and the return loss. The tested values ranged from 8.5 to 9.5 mm, with 0.5 mm increments. The best results were achieved when R_1_ = 9 mm, where the reflection coefficient reached its minimum value around 2.4 GHz.

### 4.3. Radius R_3_ of Flower Geometry

To further optimize the antenna performance, a parametric study of the radius R_2_ was also performed. Figure 6c shows the S_11_ with different R_2_ values. The obtained results indicate that the modification of R_2_ changed the impedance matching while maintaining the resonance around the desired operating frequency. Among the investigated values, R_2_ = 3 mm achieved the best S_11_, which indicated an improved impedance matching compared with the other configurations.

### 4.4. Partial Ground Length L_G_

In order to evaluate the ground plane impact on the antenna performance, a parametric study was performed by varying the length L_G_ of the partial ground. Figure 6d illustrates the obtained results. It can be noticed that the ground plane dimension played a crucial role in controlling the antenna matching and resonance characteristics. The optimal performance was obtained for L_G_ = 5 mm, where the antenna reached the minimum S_11_ of −39.56 dB at 2.4 GHz. In contrast, other values produced poorer matching and resonance deviation.

## 5. Results and Discussions

This section presents and discusses the obtained simulation and experimental results of the proposed antenna. The antenna was designed, analyzed, and validated using three simulation tools: Ansys High-Frequency Structure Simulator (HFSS Version 15.0), CST Studio Suite (CST Version 2018), and Advanced Design System (ADS Version 2020). HFSS, which is based on the finite element method (FEM), was employed for antenna design and optimization, as well as for the evaluation of the reflection coefficient (S_11_), VSWR, gain, and radiation patterns. CST Studio Suite was used to calculate the specific absorption rate (SAR) analysis. In addition, ADS was used to develop and validate the equivalent circuit model of the proposed antenna.

### 5.1. Reflection Coefficient and VSWR

**Figure 7:**
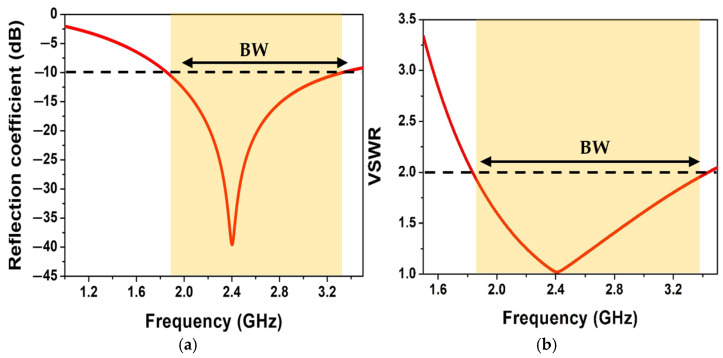
Antenna performance: (**a**) reflection coefficient; (**b**) VSWR.

Figure 7 presents the reflection coefficient and VSWR of the proposed antenna. As observed in Figure 7a, the antenna showed a resonant frequency of 2.4 GHz with a reflection coefficient minimum of −39.56 dB, which demonstrates the excellent impedance matching performance. In addition, the antenna achieved a wide operating 10 dB bandwidth covering a frequency band from 1.85 to 3.32 GHz. This obtained wide bandwidth enhances the robustness of the antenna against fabrication tolerances and frequency-detuning effects that may have an impact during practical wearable use. Moreover, the operating band fully covers the 2.4 GHz ISM band, which makes the antenna a good candidate for biomedical IoT and wireless healthcare monitoring applications. Similarly, Figure 7b presents the corresponding VSWR of the proposed antenna. The VSWR remained below 2 throughout the operating bandwidth, with a minimum value of 1.01 at 2.4 GHz, which indicates efficient power transfer and low signal reflection between the antenna and the feeding structure.

### 5.2. Gain of the Proposed Antenna

**Figure 8:**
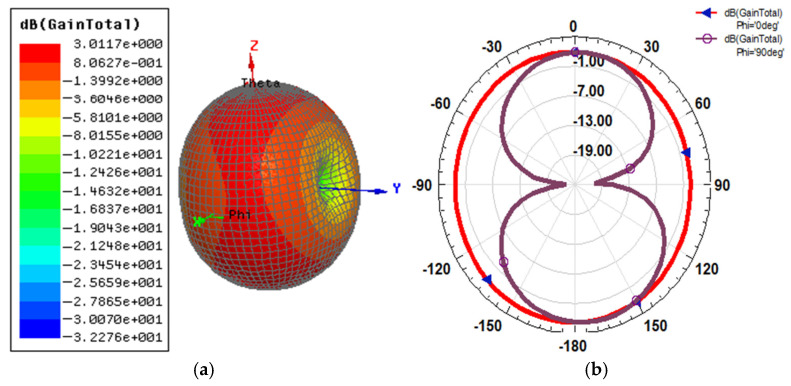
Simulated radiation patterns of the proposed antenna: (**a**) 3D radiation pattern; (**b**) 2D radiation patterns in the Φ = 0° and Φ = 90° planes.

The 2D and 3D radiation patterns of the proposed antenna are presented in Figure 8a and Figure 8b, respectively. The obtained results indicate that the antenna showed a bidirectional radiation behavior, which enabled electromagnetic wave propagation in two opposite directions. The 3D radiation pattern illustrates the spatial distribution of the radiated power, while the corresponding 2D radiation patterns in the principal planes (Φ = 0° and Φ = 90°) confirm the bidirectional nature of the antenna through the presence of two main radiation lobes. Furthermore, the radiation patterns exhibit a nearly symmetric distribution in both planes, which indicates stable radiation characteristics. The proposed antenna achieved a maximum gain of 3.01 dB, demonstrating adequate radiation performance for short-range biomedical IoT and wearable healthcare monitoring applications.

### 5.3. Simulation near to Human Body

**Figure 9:**
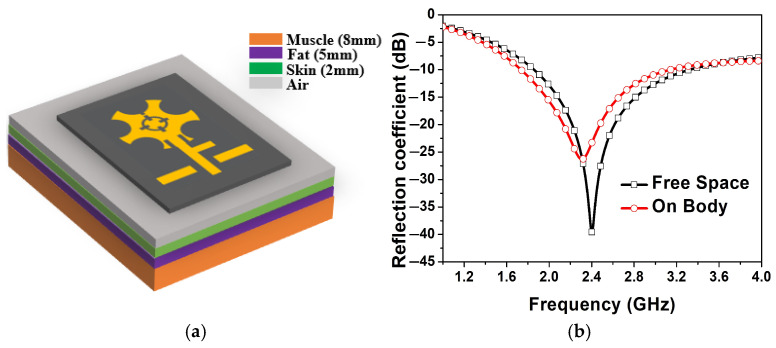
Wearable simulation: (**a**) body phantom; (**b**) simulated S_11_.

In order to evaluate the performance of the proposed antenna for biomedical applications, an on-body simulation was performed by placing the antenna in proximity to a human body phantom composed of three biological layers, skin, fat, and muscle, as illustrated in Figure 9a. The corresponding S_11_ is presented in Figure 9b. The obtained results demonstrate that the proposed antenna maintained good impedance matching in the presence of the human body environment. A reflection coefficient of −26.33 dB was achieved at the operating frequency, confirming the suitability of the proposed antenna for wearable and biomedical wireless communication applications.

### 5.4. Specific Absorption Rate (SAR)

**Figure 10:**
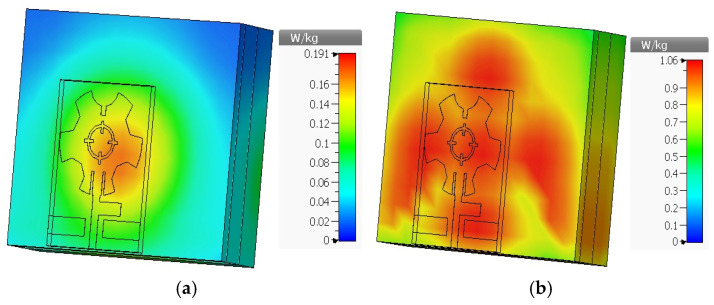
SAR of the proposed antenna: (**a**) 1 g; (**b**) 10 g.

A specific absorption rate (SAR) analysis was conducted in order to evaluate the safe use of the proposed antenna for wearable applications. The obtained SAR values for both 1 g and 10 g tissue averaging masses remained below the IEEE and ICNIRP recommended safety limits, with values of 0.19 w/kg and 1.06 w/kg, as presented in Figure 10a,b, which confirms that the proposed antenna can be safely employed for on-body biomedical communication and healthcare monitoring systems.

### 5.5. Fabricated Antenna and Measured Results

**Figure 11:**
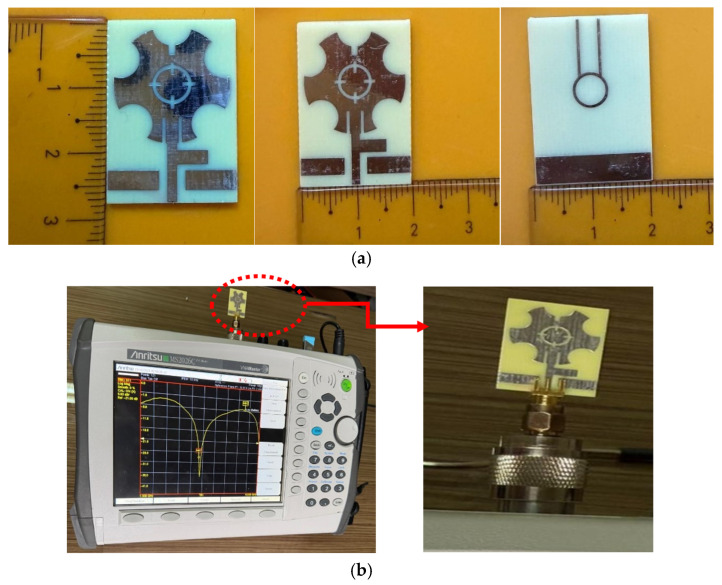
(**a**) Fabricated prototype; (**b**) vector network analyzer.

**Figure 12:**
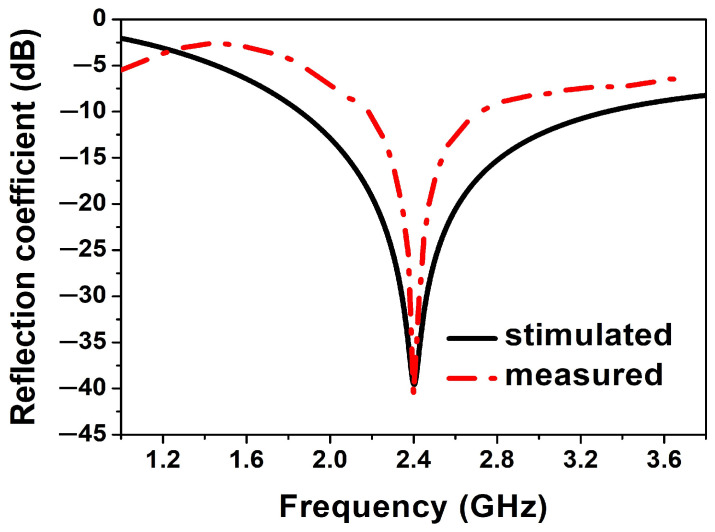
Proposed antenna: simulated and measured S_11_.

To validate the simulated results, the designed antenna was fabricated as presented in Figure 11. The fabricated prototype was measured using a vector network analyzer (VNA (Anritsu Company, Morgan Hill, CA, USA)) to characterize the reflection coefficient. The measured results illustrated in Figure 12 show good agreement with the simulation, with a measured S_11_ of −40.01 dB. The small observed variations were mainly caused by fabrication tolerances and connector losses. These results confirm the effectiveness of the proposed antenna for practical biomedical wireless communication and wearable IoT applications.

**Figure 13:**
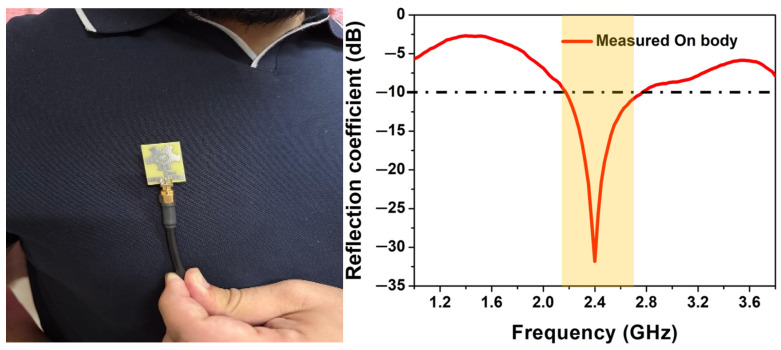
Measured S_11_ on body.

To evaluate the performance of the proposed antenna in realistic wearable cases, on-body measurements were performed by placing the antenna directly on the human chest, as presented in Figure 13. The obtained results demonstrate that the antenna preserved stable operating behavior even when we placed it in proximity to biological tissues. A reflection coefficient of −31.80 dB was achieved under on-body conditions, which indicates excellent impedance matching and limited degradation compared with free-space operation. These results confirm the robustness, reliability, and suitability of the proposed antenna for wearable biomedical applications.

## 6. Experimental Biomedical IoT Validation

To experimentally validate the proposed antenna in a real biomedical wireless communication environment, an IoT-based healthcare monitoring system was implemented using an ESP32 microcontroller and a MAX30100 biomedical sensor. The ESP32 is a low-cost microcontroller with integrated Wi-Fi capabilities widely employed in IoT applications for wireless data processing and transmission. The MAX30100 is an integrated pulse oximetry and heart-rate-monitoring sensor capable of measuring blood oxygen saturation (SpO_2_) and pulse rate. The RSSI measurements were first performed using the ESP32 module alone as a reference case and then repeated after integrating the proposed patch antenna to evaluate the improvement in wireless communication performance. The measurements were conducted in a free-space laboratory environment to ensure controlled and repeatable experimental results. The evaluation was carried out over a short-range distance varying from 1 m to 5 m, which corresponds to realistic indoor and wearable biomedical IoT scenarios, such as patient-to-gateway communication in healthcare monitoring applications. The received signal strength indicator (RSSI) was recorded at each distance for both configurations, as summarized in Table 2. The results demonstrate that the proposed antenna consistently improved the RSSI values compared with the ESP32 module alone, indicating enhanced signal strength and more stable wireless connectivity. Meanwhile, the MAX30100 sensor was used to acquire physiological parameters, such as heart rate and oxygen saturation (SpO_2_), while the ESP32 processed the collected data and transmitted them wirelessly through the proposed antenna.

**Figure 14:**
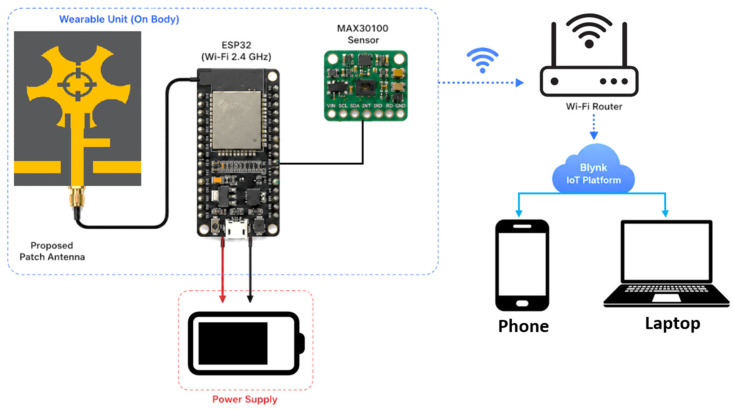
Proposed biomedical IoT monitoring system.

The experimental setup is presented in Figure 14, and, as previously described, the wearable unit integrates the proposed patch antenna, an ESP32 microcontroller, and a MAX30100 sensor. The sensor acquires the heart rate (HR) and blood oxygen saturation (SpO_2_), while the ESP32 processes the collected data and wirelessly transmits them through the proposed 2.4 GHz antenna via a Wi-Fi router to the Blynk IoT platform, enabling real-time monitoring through both smartphone and laptop interfaces. A portable power supply is used to ensure autonomous operation of the wearable device. This architecture allows continuous health monitoring and provides users with immediate access to their physiological data, while also enabling remote healthcare supervision and early detection of potential health abnormalities.

**Figure 15:**
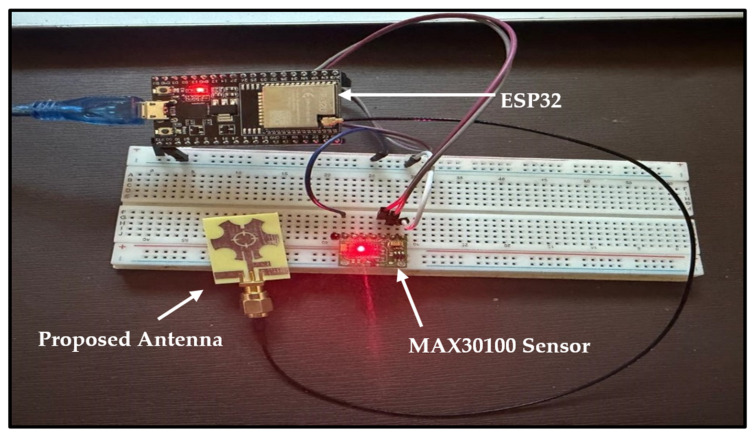
Implemented biomedical IoT monitoring system.

**Figure 16:**
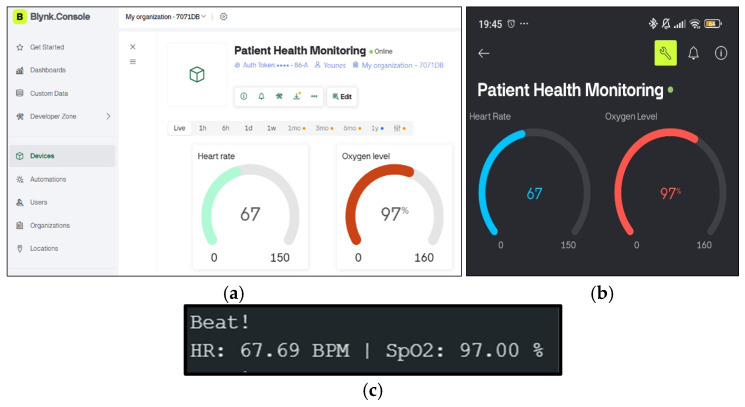
Real-time monitoring results: (**a**) on laptop, (**b**) on phone, and (**c**) serial monitor.

Figure 15 presents the hardware implementation of the proposed biomedical IoT system. The setup consists of the fabricated patch antenna, an ESP32 development board, and a MAX30100 sensor module assembled on a breadboard. The fabricated antenna is connected to the ESP32 module through an SMA cable, while the MAX30100 sensor is interfaced with the ESP32 via jumper wires. The ESP32 serves as the main processing and communication unit, whereas the MAX30100 is used as the physiological sensing module. The breadboard is employed to facilitate the electrical connections between the different components of the prototype. The obtained real-time monitoring results, including heart rate and oxygen saturation (SpO_2_) measurements displayed through the Blynk platform on both smartphone and personal computer interfaces, are presented in Figure 16. The obtained results are summarized in Table 3. The successful wireless transmission and stable monitoring performance confirm the suitability of the proposed antenna for wearable biomedical IoT and remote healthcare monitoring applications.

Table 4 presents a comparison of our work with previous published works; the comparison includes some important factors, including size, operating frequency, bandwidth, S_11_ and target application. The obtained results demonstrate that the proposed antenna achieves competitive performance while maintaining a compact structure suitable for biomedical IoT and wireless healthcare monitoring applications.

## 7. Conclusions and Future Scope

This paper presents a compact circular slotted patch antenna with a defected ground plane for biomedical applications. The antenna demonstrated enhanced performance, including a reflection coefficient of −39.56 dB and a VSWR of 1.01, which confirmed the high level of impedance matching. The suggested structure achieved a good radiation behavior with a maximum realized gain of 3.01 dB. A SAR study was conducted to confirm the safety of the antenna in a real wearable scenario. In addition, the antenna was fabricated, and the obtained measured results aligned with the simulated results, which confirmed the robustness of our structure. In order to evaluate the proposed antenna in a real biomedical application, the fabricated antenna was integrated with an ESP32 module and biomedical sensors for real-time wireless physiological data transmission. The experimental IoT-based validation demonstrated stable communication performance and efficient wireless data transmission capability, highlighting the potential of the proposed antenna for future wearable healthcare and remote patient monitoring systems. Future work will focus on multi-sensor integration and link budget analysis to further improve the reliability and applicability of the proposed biomedical monitoring system.

Future work will focus on enhancing the proposed biomedical monitoring platform through the integration of multiple physiological sensors, such as temperature, heart rate, blood oxygen saturation (SpO_2_), and blood pressure sensors, to enable comprehensive real-time health monitoring. Further investigations will be conducted to evaluate communication performance over extended transmission distances and in various indoor and outdoor environments, accompanied by a detailed link budget analysis to assess system reliability and coverage. In addition, on-body measurements considering different body locations and user activities will be performed to validate the antenna performance in realistic wearable scenarios. The development of flexible and textile-based antenna implementations will also be explored to improve user comfort and wearable integration. Finally, the integration of the proposed antenna with advanced IoT healthcare platforms, cloud-based monitoring systems, and intelligent data-processing techniques will be investigated to support next-generation remote patient monitoring and personalized healthcare applications.

## Figures and Tables

**Table 1:** Dimensions of the antenna.

Parameter	Value (mm)	Parameter	Value (mm)	Parameter	Value (mm)	Parameter	**Value (mm)**
W	20	R_3_	3	W_T_	0.5	L_A_	2.2
L	28	W_F_	1.5	L_T_	4	W_B_	0.5
H	1.6	L_F_	9.2	W_X_	0.5	L_B_	9.7
R_1_	9	W_E_	8	L_X_	2	R_4_	3
R_2_	2.5	L_E_	3	W_A_	5	L_G_	5

**Table 2:** ESP32 RSSI with and without antenna.

Distance	RSSI ESP32 (dBm)		
	Without Antenna	With Antenna	Improvement
1 m	−70	−23	47
2 m	−75	−31	44
3 m	−79	−38	41
4 m	−81	−41	40
5 m	−85	−45	40

**Table 3:** Example of the received data.

Test No.	Heart Rate (bpm)	SpO_2_ (%)
Test 1	67	97
Test 2	68	96
Test 3	69	97
Test 4	71	98
Test 5	68	99
Test 6	67	97
Average	68.3	97.3

**Table 4:** Comparison of our proposed patch antenna with previous works.

Ref.	Size (mm^2^)	Substrate	Frequency (GHz)	Bandwidth (GHz)	S_11_ (dB)	Application
[22]	29 × 7	FR4	2.4	2.18–3.09	−25.79	Biomedical
[23]	25 × 20	Polyimide	2.46	2.45–2.48	−20.58	Biomedical
[24]	45 × 30	FR4	2.4	2.20–2.79	−62.76	Biomedical
[25]	26 × 22	Polyimide	2.46	2.38–2.77	−33.9	Biomedical
[26]	24 × 22	Polyimide	2.41	2.01–2.82	−25.34	Biomedical
[27]	10 × 14	Ceramic	2.45	180 MHz	−27.01	Biomedical
[Prop]	28 × 20	FR4	2.4	1.85–3.32	−39.56	Biomedical

## Data Availability

The original contributions presented in this study are included in the article. Further inquiries can be directed to the corresponding author.

## Abbreviations

The following abbreviations are used in this manuscript:

IoT	Internet of Things
VNA	Vector Network Analyzer
SAR	Specific Absorption Rate
DGS	Defected Ground Structure
ESP32	Espressif 32-Bit Microcontroller
RSSI	Received Signal Strength Indicator
SpO_2_	Peripheral Capillary Oxygen Saturation
HR	Heart Rate
ADS	Advanced Design System
HFSS	Ansys High-Frequency Structure Simulator
